# The relationship between chronic pain and health-related quality of life: the mediating roles of frailty and depression in chronic disease patients

**DOI:** 10.3389/fpubh.2025.1658008

**Published:** 2025-09-01

**Authors:** Yongli Shi, Mengyuan Xu, Wenqiang Yin, Ziyuan Li, Ping Dong, Xianqi Zhang, Haoqi Li, Xianglan Zhuge, Xiaona Li, Min Gao, Dongping Ma, Kui Sun, Haihong Cao, Zhongming Chen

**Affiliations:** ^1^School of Management, Shandong Second Medical University, Weifang, China; ^2^Weifang People’s Hospital, Shandong Second Medical University, Weifang, China

**Keywords:** frailty, depression, chronic disease management, chronic pain, chain mediation

## Abstract

**Introduction:**

Chronic pain (CP) is a prevalent comorbidity in patients with chronic diseases, yet the relationship between CP and health-related quality of life (HRQoL) remain unclear, particularly through the mediating roles of frailty and depression.

**Methods:**

We conducted a cross-sectional survey in two provinces (eastern and central China) from October 2024 to January 2025, enrolling 3,094 patients with chronic diseases. HRQoL was assessed using the EQ-5D scale, while CP status was determined through structured logic-based questions. Frailty was evaluated using the FRAIL scale, and depressive symptoms were measured with the CES-D10. Spearman’s correlation analysis was performed to assess associations among CP, frailty, depression, and HRQoL. A chain mediation model (PROCESS 4.1, Model 6) was constructed, and mediation effects were tested using a bootstrap approach with 5,000 resamples.

**Results:**

Frailty and depression exhibited significant mediating effects in the relationship between CP and HRQoL. The indirect effects of frailty and depression on HRQoL were −0.0747 (95% CI: −0.0881, −0.0618) and −0.0211 (95% CI: −0.0297, −0.0135). Additionally, a significant chain mediation effect was observed (−0.0192, 95% CI: −0.0242, −0.0145). The indirect effect of frailty and depression accounted for 34.09% of the association between CP and HRQoL (total effect: −0.1150, 95% CI: −0.1305, −0.0999).

**Conclusion:**

The study findings demonstrated that frailty and depression serve as significant chain mediators in the relationship between CP and diminished HRQoL. Measures should be taken to reduce the incidence and severity of CP in patients with chronic diseases, improve frailty and depression, and thus improve HRQoL.

## 1 Introduction

With sustained improvements in global population health and significant gains in life expectancy, chronic diseases have emerged as the leading disease burden worldwide. Recent data from the World Health Statistics 2024 revealed that chronic diseases accounted for 65.3% of global mortality in 2021 (1). National epidemiological studies indicated an even more pronounced burden in China, where chronic diseases contributed to over 80% of total deaths (2). Chronic disease patients experience heavier health burdens than healthy individuals, including more severe pain, fatigue, psychological distress, functional impairments, and reduced Health-related quality of life (HRQoL) (3).

HRQoL is a multidimensional concept reflecting an individual’s subjective perception of physical, psychological, and social well-being within their cultural and personal context (4, 5). For chronic disease patients (CDPs), who often face prolonged illness, poor HRQoL—driven by physical limitations and reduced daily functioning—is common (6–8). Understanding these determinants is key to designing targeted interventions that improve well-being, uphold dignity, and mitigate the societal impacts of aging.

Chronic pain (CP), defined as pain lasting over 3 months (9), is the world’s third most prevalent health condition after cardiovascular disease and cancer (10–12). Globally affecting about 33% of people (13), China has over 300 million cases, with 10–20 million new annual diagnoses (14). CP frequently coexists with chronic diseases, often causing more severe pain (15). In China, 63.4% of middle-aged/older adults report chronic disease-related pain (16), with prevalence rising alongside chronic conditions (17, 18). Persistent pain leads to mobility issues, higher fall risk, depression, psychological distress, and reduced HRQoL (19, 20). While the CP-HRQoL link is established, underlying mechanisms need further study.

Frailty, characterized by reduced physiological resilience and multisystem decline (21), is prevalent among CDPs and ranks as their third most common comorbidity (22, 23). Frailty prevalence varied substantially among chronic conditions: exceeding 30% in chronic pancreatitis patients (24), reaching 61.8% in type 2 diabetes populations (25), and tripling morbidity risk in older adults cardiovascular patients versus controls (26). Growing evidence showed frailty was a major consequence of CP (27–30). Research demonstrates CP patients show elevated frailty rates compared to pain-free individuals (31), with frailty substantially increasing risks for falls, hospitalization, disability, and mortality (32, 33). Frail older adults also exhibit higher susceptibility to physiological dysregulation, depression, and cognitive decline (34–36) key determinants of health span and quality of life in aging populations (37–40).

Depression, characterized by persistent low mood and anhedonia, affects 3.8% of the global population and worsens outcomes for chronic diseases (41–43). Prevalence is particularly high in CDPs—38.3% in rheumatoid arthritis (44) 19.97% in U. S. resettlement communities (45), and 41.3% in eastern China (46). Among CP patients, 30–50% experience depressive symptoms (47, 48). Depression accelerates physical decline through fatigue and sleep disturbances while causing cognitive dysfunction and social withdrawal (49). As the world’s most common mental disorder, it significantly impairs HRQoL (50–53).

While prior research has established connections between CP, frailty, depression and HRQoL, the specific mediating pathways remain unclear. This study investigated how frailty and depression transmit CP’s impact on HRQoL in chronic disease patients. We propose four hypotheses:

*Hypothesis 1*: CP will demonstrate a significant negative association with HRQoL in patients with chronic diseases.

*Hypothesis 2*: Frailty will significantly mediate the relationship between CP and reduced HRQoL.

*Hypothesis 3*: Depression will significantly mediate the association between CP and impaired HRQoL.

*Hypothesis 4*: Frailty and Depression will operate as sequential mediators in the pathway between CP and reduced HRQoL.

## 2 Methods

### 2.1 Sample selection

This study utilized data from a cross-sectional survey conducted between October 2024 and January 2025 across two Chinese provinces representing eastern and central regions. The survey employed a multi-stage stratified random sampling approach to ensure population representativeness. The first stage was to select sample cities according to the low, middle, and high levels of economic development. The second stage was to select 3 districts (counties) randomly in each city and exact 4 townships (streets) in each selected district (county), and then choose 6 villages (communities) from each township (street). The third stage was to choose 23 patients with chronic diseases randomly from each village (community) for the survey. Finally, samples were screened for inclusion in the analysis according to the needs of the study.

The inclusion criteria for this study were the completion of all frailty, depression scales, and HRQoL assessments during follow-up. Exclusion criteria for this study: (1) Patients with severe hearing, visual, and communication (i.e., language expression) disorders, mental illness, and serious physical illness; (2) Subjects who gave incomplete answers. Finally, 3,094 people were included in the analysis. Written informed consent was obtained from each participant before the survey.

### 2.2 Chronic pain

Chronic pain (CP) was defined as pain that persists or recurs for more than 3 months (9). CP status was ascertained through a validated algorithmic questionnaire employing sequential logic-based screening items. In the questionnaire, “Do you have CP?”? No/Yes “If the respondent answers” No, the respondent is classified as “painless”. If the respondent has CP, further ask “What do you think is the level of CP?.” Respondents were asked to rate the level of CP as “not severe at all,” not too severe, “fair,” moderately severe, “and” very severe, “on a numerical scale of 1 to 5. If the respondent did not have CP, a score of 0 was given. The higher the score, the more severe the CP.

### 2.3 Health-related quality of life

EQ-5D (Euro Qol Five Dimensions Questionnaire) was used to measure the HRQoL. HRQoL was quantified using the EQ-5D-5L instrument, with utility scores calculated according to the Chinese value set (coefficient-based scoring algorithm) (54).

### 2.4 Frailty

The FRAIL scale was used to assess the frailty status of the participants. The scale consists of 5 items with a full score of 5. The higher the score, the more severe the frailty status (55).

### 2.5 Depression

The CES-D10 scale measured depression. The scale contains 10 items. The overall score of the scale ranges from 0 to 30. The higher the score, the higher the degree of depression (56).

### 2.6 Covariates

Based on prior evidence (52–57), we adjusted for established HRQoL covariates including socio-demographics, health behaviors, and physical health status in our analyses. (1) Socio-demographic characteristics: a self-made demographic questionnaire was used, including age (years), gender (male, female), marital status [married, not married], living status (living alone, not living alone), education level (primary school and below, junior high school, senior high school and technical secondary school, junior college and above), working status (employed, not employed). (2) Health behaviors include smoking status (smoking, smoking before, not smoking now, never smoking), drinking status (drinking, drinking before, not drinking now, never drinking), physical exercise (never doing physical exercise, 1–2 times/week, 3–5 times/week, more than 5 times/week), social activities (no, yes). (3) Physical health status includes chronic disease status [(number of diseases: 1, 2, ≥3), course of disease (years)]. Chronic disease status was defined as the presence of a chronic disease that the subject was considered to have.

### 2.7 Statistical analysis

First, the mean (*SD*) of the continuous variable and the frequency (*%*) of the categorical variable were applied to report the sociodemographic characteristics of the sample. Normality of CP, HRQoL, debilitation, and depression was examined by the Kolmogorov–Smirnov normality test. The results showed that not all variables were normally distributed (*p* < 0.001). The Mann–Whitney test (*U* test) and the Kruskal-Wallis test (*H* test) were used to compare the EQ-5D utility values of patients with chronic diseases with different socio-demographic characteristics. Because of the large sample size, Dunn’s test was used for further multiple comparisons. Bivariate associations among CP, HRQoL, frailty, and depression were assessed using Spearman’s rank correlation coefficients. The items of CP, HRQoL, frailty, and depression scales were tested for common method bias using the Harman univariate test.

To demonstrate whether there is a series of multiple mediating effects, such as debilitation and depression, between CP and HRQoL, we used the SPSS macroprocess program (Model 6) designed by Hayes (58) to complete the data analysis. A *p* value of less than 0.05 was considered statistically significant. The statistical significance of the mediating effect was tested using the Bootstrap method, and estimates of the mediating effect were generated, and bias-corrected 95% confidence intervals (CIs) were calculated by a resampling procedure with 5,000 samples. Two-tailed *p* < 0.05 was considered statistically significant.

## 3 Result

### 3.1 Basic information of participants

Table 1 showed the demographic characteristics of the study participants. Of the 3,094 participants, 62.09% were female, 71.68% were patients with chronic diseases over 70 years old, their average age was (70.00 ± 8.32) years old, and most of the participants were currently married (73.92%). Overall, the education level of the participants was generally low, with 63.87% of them having an education level of primary school or below. Additionally, 35.42% of the participants had one chronic disease, while 64.58% were comorbid with multiple chronic diseases. 29.64% of the participants exhibited frailty. The prevalence of CP was 63.45%, with the majority (55.82%) having experienced it for 10 years or less. The health utility value of EQ-5D was higher in males, younger, married, more educated, employed, less chronic diseases, shorter disease course, smoking, drinking, more physical exercise, not living alone, less CP, and less debilitating participants (*p* < 0.001). See Table 1 for details.

**Table 1:** Distribution of EQ-5D health utility values with different demographic characteristics (*N* = 3,094).

Variable		EQ-5D ( $\bar{x}\pm s$ )	*n* (%)	*H/U*	*p*-value
Gender	Male	0.863 ± 0.195	1,173 (37.91%)	969069.500*^U^*	<0.001
	Female	0.832 ± 0.199	1,921 (62.09%)		
Age	<60	0.910 ± 0.152	386 (12.48%)	98.859*^H^*	<0.001
	60–64	0.866 ± 0.180	390 (12.61%)		
	65–69	0.853 ± 0.185	486 (15.71%)		
	70–74	0.831 ± 0.203	815 (26.34%)		
	75–79	0.818 ± 0.207	681 (22.01%)		
	>80	0.810 ± 0.229	336 (10.86%)		
Marital status	Married	0.821 ± 0.209	2,287 (73.92%)	830235.500*^U^*	<0.001
	Others^a^	0.852 ± 0.193	807 (26.08%)		
Education	Primary school and below	0.829 ± 0.204	1,976 (63.87%)	53.722*^H^*	<0.001
	Middle school	0.868 ± 0.181	836 (27.02%)		
	High school and above	0.873 ± 0.192	282 (9.11%)		
Work status	Working	0.875 ± 0.145	530 (17.13%)	630366.000*^U^*	0.008
	Not working	0.837 ± 0.207	2,564 (82.87%)		
Number of chronic diseases	1	0.898 ± 0.153	1,096 (35.42%)	253.321*^H^*	<0.001
	2	0.847 ± 0.193	1,018 (32.90%)		
	≥3	0.779 ± 0.228	980 (31.67%)		
Course of disease	≤10	0.870 ± 0.172	1,727 (55.82%)	72.104*^H^*	<0.001
	11–29	0.812 ± 0.224	1,140 (36.85%)		
	≥30	0.808 ± 0.212	227 (7.34%)		
Smoking status	Smoking	0.872 ± 0.180	502 (16.23%)	24.373*^H^*	<0.001
	Suck before, not now	0.828 ± 0.226	307 (9.92%)		
	Never	0.839 ± 0.197	2,285 (73.85%)		
Alcohol consumption	Drinking	0.874 ± 0.182	429 (13.87%)	21.401*^H^*	<0.001
	Used to drink, not now	0.832 ± 0.213	258 (8.34%)		
	Never	0.840 ± 0.199	2,407 (77.80%)		
Physical exercise	Never	0.749 ± 0.271	623 (20.14%)	122.494*^H^*	<0.001
	1–2 times/week	0.854 ± 0.180	316 (10.21%)		
	3–5 times/week	0.859 ± 0.165	467 (15.09%)		
	More than 5 times/week	0.873 ± 0.164	1,688 (54.56%)		
Social activities	Yes	0.854 ± 0.177	2,230 (72.08%)	928505.500*^U^*	0.113
	No	0.818 ± 0.242	864 (27.93%)		
Living arrangement	Alone	0.823 ± 0.195	663 (21.43%)	713003.500*^U^*	<0.001
	Not living alone	0.849 ± 0.198	2,431 (78.57%)		
Chronic pain status	Painless	0.923 ± 0.159	1,131 (36.56%)	811.659*^H^*	<0.001
	Not serious at all	0.920 ± 0.096	49 (1.58%)		
	Not too serious	0.875 ± 0.131	441 (14.25%)		
	General	0.844 ± 0.154	505 (16.32%)		
	More serious	0.762 ± 0.201	846 (27.34%)		
	Very serious	0.532 ± 0.334	122 (3.94%)		

*H* is the statistic of the Kruskal-Wallis test; *U* is the statistic of the Mann–Whitney test.

a Others includes single, divorced and widowed.

### 3.2 Common deviation test

Harman’s single-factor test method was used to test the input common deviation of variables in each scale (59). The results showed that there were 6 factors with characteristic roots greater than 1 extracted by the factors. The explanation rate of the first common factor was 29.40%, which was less than the critical value of 40.00%, indicating that the common deviation of this study was not significant and the research results are credible.

### 3.3 Variable description statistics and correlations

The scores of frailty, depression, and HRQoL were (1.502 ± 1.322), (18.344 ± 6. 393), and (0.844 ± 0. 198), respectively. The mean and difference of CP, frailty, depression, and HRQoL, and the Spearman correlation test results between the variables are shown in Table 2. Results showed that CP was negatively associated with HRQoL (*r* = −0.509, *p* < 0.001) and positively associated with frailty (*r* = 0.383, *p* < 0.001) and depression (*r* = 0.281, *p* < 0.001). Frailty was positively associated with depression (*r* = 0.434, *p* < 0.001) and negatively associated with HRQoL (*r* = −0.513, *p* < 0.001). Depression was negatively associated with health-related life (*r* = −0.412, *p* < 0.001).

**Table 2:** Statistical description of pain, frailty, depression, and HRQoL and Spearman correlation analysis between them.

Variables	Score ( $\bar{x}\pm s$ )	Chronic pain	Frailty	Depression	HRQoL
Chronic pain	–	1.000			
Frailty	1.502 ± 1.322	0.383^***^	1.000		
Depression	18.344 ± 6.393	0.281^***^	0.434^***^	1.000	
HRQoL	0.844 ± 0.198	−0.509^***^	−0.513^***^	−0.412^***^	1.000

***p < 0.001.

### 3.4 Chain mediating effect of frailty and depression between CP and HRQoL

Using model 6 of the SPSS PROCESS V4.1 macro program, after controlling for the covariates, the 95% confidence interval of each effect was estimated by using the Bootstrap method, sampling 5,000 times, and the mediating effects of frailty and depression on CP and HRQoL were tested.

Regression showed that *R^2^* indicated that the model explained 22.46% of the variance in frailty, 23.92% of the variance in depression, and 33.74% of the variance in HRQoL. Specifically, the direct effect of CP situations on HRQoL was significant (*β* = −0.337, *p* < 0.001) before the inclusion of mediating variables. With the introduction of mediators of frailty and depression, the positive effect of CP situations on frailty g was significant (*β* = 0.300, *p* < 0.001), positive effect on depression was also significant (*β* = 0.105, *p* < 0.001), and the direct effect on HRQoL remained significant (*β* = −0.223, *p* < 0.001). This suggests that there is a partial mediating effect between frailty and depression. Frailty significantly positively affected depression (*β* = 0.318, *p* < 0.001) and negatively affected HRQoL (*β* = −0.250, *p* < 0.001). Meanwhile, depression also negatively affected HRQoL (*β* = −0.201, *p* < 0.001). This suggests that CP not only directly contributes to HRQoL but also indirectly exacerbates negative effects through chained mediating pathways of frailty and depression, as shown in Tables 3, 4.

**Table 3:** Results of multiple mediating utility tests of frailty and depression between pain and HRQoL.

Variables	Frailty		Depression		HRQoL	
	*β*	*t*	*β*	*t*	*β*	*t*
Gender	0.065	2.895^**^	0.013	0.590	0.026	1.260
Age	0.179	9.598^***^	−0.010	−0.557	−0.063	−3.583^**^
Marital status	0.025	1.035	−0.083	−3.413^**^	0.009	0.372
Education	−0.028	−1.615	−0.056	−3.267^**^	−0.021	−1.294
Work status	0.041	2.433^*^	−0.017	−0.100	0.011	0.726
Number of chronic diseases	0.118	6.876^***^	0.170	9.917^***^	−0.048	−2.928^**^
Course of disease	0.028	1.655	0.041	2.424^*^	−0.018	−1.162
Smoking status	−0.032	−1.435	−0.054	−2.467^*^	−0.011	−0.548
Alcohol consumption	−0.008	−0.389	0.034	1.641	0.003	0.157
Physical exercise	−0.102	−6.187^***^	0.006	0.362	0.157	10.246^***^
Social activities	−0.093	−5.646^***^	−0.047	−2.883^**^	−0.057	−3.730^**^
Living arrangement	−0.075	−3.108^**^	0.012	0.481	−0.029	−1.293
Chronic pain status	0.300	17.653^***^	0.105	5.966^***^	−0.223	−13.412^***^
Frailty			0.318	17.829^***^	−0.250	−14.225^***^
Depression					−0.201	−11.937^***^
*R*	0.4739		0.4890		0.5808	
*R* ^2^	0.2246		0.2392		0.3374	
*F*	68.620^***^		69.130^***^		104.476^***^	

*p < 0.05, **p < 0.01, ***p < 0.001.

**Table 4:** Analysis of the mediating effect of frailty and depression.

Pattern path	*β*	*SE*	Proportion of total effect (%)	95%CIs	
				Lower	Upper
Total effect	−0.337	0.017	100.00%	−0.371	−0.304
Direct effect	−0.222	0.017	65.91%	−0.255	−0.190
Total indirect effect	−0.115	0.008	34.09%	−0.131	−0.100
CP → frailty → HRQoL	−0.075	0.007	22.15%	−0.088	−0.062
CP → depression → HRQoL	−0.021	0.004	6.26%	−0.030	−0.014
CP → frailty → depression → HRQoL	−0.019	0.003	5.69%	−0.024	−0.015

### 3.5 Estimation of the confidence interval of the mediation effect test

**Figure 1:**
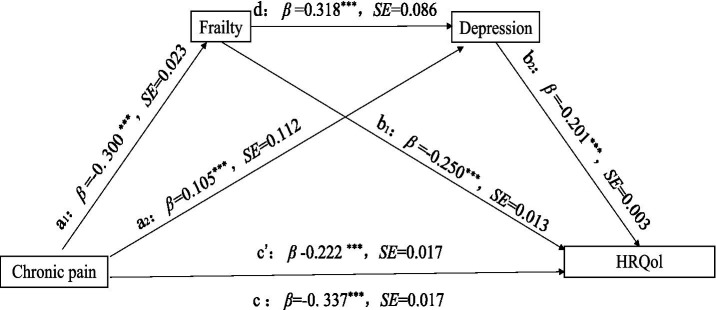
Correlation pathway diagram of frailty and depression as mediators of pain and HRQoL. ***p < 0.001, **p < 0.01, *p < 0.05. The model controls sex, age, sex, marital status, education level, work situation, number (species) of chronic diseases, course (years) of diseases, smoking and drinking, physical exercise, social activities, and living conditions.

Table 4 presented the results of the mediating effects of frailty and depression on the relationship between CP and HRQoL. None of the 95% confidence intervals of the 3 paths contained 0, indicating that the indirect effects of the 3 paths reached a significant level, as shown in Figure 1. In the indirect effect model, the total indirect effect of CP caused by frailty and depression was −0.1150 (95%CI = −0.1305 ∼ −0.0999), with 34.09% of the total effect mediated. Specifically, frailty and depression explained 22.15 and 6.26% of the total effect, respectively, and 5.69% through the frailty-mediated depression pathway.

## 4 Discussion

HRQoL serves as a crucial prognostic indicator for mortality and hospitalization risk (60), offering a comprehensive evaluation of physical, psychological, social, and life satisfaction domains (6, 7) that surpasses traditional health metrics. The WHO incorporates HRQoL in Healthy Life Years (HALE) calculations as a key health metric (61). This study pioneered the examination of frailty and depression as chain mediators in the CP-HRQoL relationship among CDPs. The results supported all hypotheses, establishing CP as a significant negative predictor of HRQoL-CDPs with greater CP severity showed markedly lower HRQoL scores, revealing a novel mechanistic framework.

These results corroborate previous findings on CP’s detrimental effect on HRQoL (62), mediated through both physiological and psychological pathways. CP commonly accompanies tissue inflammation (63), neuropathy (64), and disease progression (65), causing functional impairments like mobility limitations (66) and sleep disturbances (67) that reduce physical capacity and daily functioning. These chronic impairments foster learned helplessness and social withdrawal (68), exacerbating isolation and ultimately worsening HRQoL.

CP directly impairs HRQoL while also indirectly reducing it via frailty mediation, potentially through hypothalamic–pituitary–adrenal (HPA) axis activation. This neuroendocrine response leads to sustained hypercortisolemia, which subsequently triggers a cascade of pathophysiological consequences including immunosuppression, metabolic dysregulation, and enhanced fatigue perception. These changes accelerate frailty by impairing immunity, disrupting metabolism, and increasing disease susceptibility (69), worsening physical decline. CP-induced inflammation and catabolism (63) impair energy metabolism, promoting frailty and creating a debilitating cycle of muscle weakness and functional decline that worsens HRQoL. Alternatively, CP may exacerbate frailty through multiple interconnected pathways. First, pain-induced activity restriction can lead to musculoskeletal atrophy and progressive functional deterioration. Second, it negatively impacts treatment adherence and reduces participation in rehabilitation programs among CDPs (66). Third, CP induces systemic inflammation and metabolic dysfunction while disrupting sleep and appetite, leading to nutritional deficits. Finally, the psychological sequelae of CP, including depression and cognitive dysfunction, further compound physical decline. These multifactorial mechanisms collectively accelerated the frailty trajectory in affected individuals. The progressive loss of self-management capacity (28–30, 70) critically exacerbates frailty and impairs HRQoL, creating a vicious cycle where declining function further reduces health-preserving behaviors and accelerates physical deterioration.

CP negatively impacted HRQoL in CDPs through depression mediation, aligning with known CP-depression associations (71). As a chronic stressor, CP consistently correlates with psychological distress and depression risk (72, 73). Neurobiological mechanisms include CNS pathway dysregulation, particularly altered opioid/dopamine receptor function (73, 74), while pro-inflammatory cytokines may disrupt 5-HT and dopamine synthesis (75), impairing emotional regulation and increasing depression susceptibility. CP reduced patients’ daily activities and social participation, increasing loneliness and depression risk (76). Depression worsened treatment adherence and well-being while impairing functional capacity and social adaptation, creating perceived loss of control that further diminished self-efficacy and HRQoL.

This study demonstrated that frailty and depression sequentially mediate 34.09% of CP’s total negative effect on HRQoL in chronic disease patients, representing a substantial mediation pathway. CP-induced functional decline (21) establishes frailty and depression as key sequential mediators in reducing HRQoL. Physical decline fosters depression through psychological vulnerability (77, 78) and subsequent physiological dysregulation (79). These pathophysiological alterations subsequently impaired subjective well-being, thereby accelerating the progressive deterioration of HRQoL in affected patients.

## 5 Conclusion

The study revealed that CP not only directly reduces HRQoL in patients with chronic diseases, but also indirectly impacts it through frailty and depression. These factors act as both individual and sequential mediators, forming a significant mediation pathway. This indirect pathway explains 34.09% of CP’s total effect on HRQoL. While the findings provide important insights, the cross-sectional design limits causal inferences and cannot address potential bidirectional relationships between mediators, highlighting the need for future longitudinal studies to verify these pathways and further elucidate the complex interrelationships between CP, frailty, depression and HRQoL in this vulnerable population.

## Data Availability

The original contributions presented in the study are included in the article/supplementary material, further inquiries can be directed to the corresponding authors.
